# Referring and Specialist Physician Gender and Specialist Billing

**DOI:** 10.1001/jamanetworkopen.2023.28347

**Published:** 2023-08-25

**Authors:** Nadine Chami, Sharada Weir, Shaun A. Shaikh, Lyn M. Sibley, Sarah Simkin, James G. Wright, Jasmin Kantarevic

**Affiliations:** 1 Economics, Policy & Research Department, Ontario Medical Association, Toronto, Ontario, Canada; 2 Abt Associates, Rockville, Maryland; 3 Now in private practice, Austin, Texas; 4 Canadian Centre for Health Economics, University of Toronto, Toronto, Ontario, Canada; 5 Canadian Health Workforce Network, Ottawa, Ontario, Canada; 6 Department of Family Medicine, University of Ottawa, Ottawa, Ontario, Canada; 7 Department of Surgery, University of Toronto, Toronto, Ontario, Canada; 8 Institute of Health Policy, Management and Evaluation, University of Toronto, Toronto, Ontario, Canada; 9 Deparment of Economics, University of Toronto, Toronto, Ontario, Canada; 10 Insitute of Labor Economics, Deutsche Post Foundation, Bonn, Germany

## Abstract

**Importance:**

While a gender pay gap in medicine has been well documented, relatively little research has addressed mechanisms that mediate gender differences in referral income for specialists.

**Objective:**

To examine gender-based disparities in medical and surgical specialist referrals in Ontario, Canada.

**Design, Setting, and Participants:**

This cross-sectional study included referrals for specialist care ascertained from Ontario Health Insurance Plan physician billings for fiscal year 2018 to 2019. Participants were specialist physicians who received new patient consultations from April 1, 2018, to March 31, 2019, and the associated referring physicians. Data were analyzed from April 2018 to March 2020, including a 12-month follow-up period.

**Exposures:**

Specialist and referring physician gender (female or male).

**Main Outcomes and Measures:**

Revenue per referral was defined based on an episode-of-care approach as total billings for a 12-month period from the initial consultation. Mean total billings for female and male specialists were compared and the differential divided into the portion owing to referral volume vs referral revenue. Difference-in-differences multivariable regression analysis was used to estimate gender-based differences in revenue per referral. For each referring physician, gender-based differences in referral patterns were examined using case-control analysis, in which specialists who received a referral were compared with matched control specialists who did not receive a referral. This analysis considered the gender of the specialist and concordance between the gender of the referring physician and specialist, among other characteristics.

**Results:**

Of 7 621 365 new referrals, 32 824 referring physicians, of whom 13 512 (41.2%) were female (mean [SD] age, 46.3 [11.6] years) and 19 312 (58.8%) were male (mean [SD] age, 52.9 [13.5] years), made referrals to 13 582 specialists, of whom 4890 (36.0%) were female (mean [SD] age, 45.6 [11.0] years) and 8692 (64.0%) were male (mean [SD] age, 51.8 [13.0] years). Male specialists received more mean (SD) referrals than did female specialists (633 [666] vs 433 [515]), and the mean (SD) revenue per referral was higher for males ($350 [$474]) compared with females ($316 [$393]). Adjusted analysis demonstrated a −4.7% (95% CI, −4.9% to −4.5%) difference in the revenue per referral between male and female specialists. Multivariable regression analysis found that physicians referred more often to specialists of the same gender (odds ratio, 1.04; 95% CI, 1.03-1.04) but had higher odds of referring to male specialists (odds ratio, 1.10; 95% CI, 1.09-1.11).

**Conclusions and Relevance:**

In this cross-sectional study of the gender pay gap in specialist referral income, the number and revenue from referrals received differed by gender, as did the odds of receiving a referral from a physician of the same gender. Future research should examine the effectiveness of different policies to address this gap, such as a centralized, gender-blinded referral system.

## Key Points

**Question:**

Are there associations between the gender of referring and specialist physicians and specialist billing?

**Findings:**

In this cross-sectional study of 7.6 million new referrals made by 32 824 referring physicians to 13 582 specialists in Ontario, Canada, female specialists earned 4.7% less in revenue per referral than did male specialists. A male physician referring to a female specialist was associated with a lower referral revenue, and referring physicians had higher odds of referring to specialists of the same gender.

**Meaning:**

Results of this study suggest that gender-based differences in referral income for specialists may persist absent policy intervention.

## Teaser

This cross-sectional study examines differences in referral-based billing by the gender of referring and specialist physicians in Ontario, Canada.

## Introduction

Gender pay inequity in medicine has been documented across jurisdictions, with female physicians earning consistently less than male physicians.^1,2,3,4,5,6,7,8,9,10,11,12,13,14^ One important mechanism discussed in the literature is specialist income and the role of gender-based referral bias. Anecdotal evidence has been documented about gender-based referral biases leading to significant pay gaps, including referrals of lower quality and revenue.^15^ Female specialists have been reported to perform less complex cases and work fewer days per period than males.^16,17,18^ A longitudinal study from Ontario, Canada, found that female surgeons received fewer referrals than male surgeons throughout their career pathway irrespective of experience and that this did not self-correct over time.^19^ Studies in the US have found evidence that physicians tend to refer to specialists of the same gender.^20,21^ One study found that female surgeons experienced more severe repercussions from referring physicians after negative surgical outcomes than male surgeons (eg, a patient death was associated with fewer and lower-revenue referrals for female surgeons) and that a negative experience with 1 female physician tended to reduce the likelihood of new referrals to other female surgeons within the same specialty.^22^ Types of gender bias hypothesized to mediate referral differences include bias from peers, hospital staff, surgical leadership, and patients.^23^

In this study, we examined the gender-based differences in referral patterns and earnings of specialists in Ontario, Canada. In Ontario’s publicly funded system, referrals are typically generated by an initial request from a physician seeking the professional opinion of a consultant physician, who is usually a specialist.^24^ Specialists not receiving a referral earn reduced compensation. While Ontario’s system may differ from other jurisdictions in important respects, the results of this study may be relevant anywhere biases in referral patterns are of concern for pay equity.

To study this hypothesis, we examined differences in referral-based pay by the gender of referring physicians and specialists. We contributed to the policy-relevant literature by investigating the relative importance of gender-based differences in referral income based on the number of referrals and revenue and analyzed factors associated with both.

## Methods

This cross-sectional study examined gender-based differences in specialist referrals using data from a publicly funded universal health care system in Ontario, Canada. We adhered to the Strengthening the Reporting of Observational Studies in Epidemiology (STROBE) reporting guideline for cross-sectional studies. Both ethics approval and informed consent were not required because the study used deidentified administrative health data obtained from the Ontario Ministry of Health under an agreement with the Ontario Medical Association, and research was initially conducted as part of business operations.

### Data Sources

Referral data and physician specialty were obtained from Ontario Health Insurance Plan (OHIP) claims data, the repository of all fee-for-service physician billings submitted and paid under the province’s single-payer health care system (eFigure 1 in Supplement 1). Data on physician and patient characteristics were identified from the Corporate Provider Database and the Registered Persons Database, respectively. Leaves of absence were identified from the Pregnancy and Parental Leave Benefit Program database. Patient-level clinical complexity was estimated using the Canadian Institute for Health Information’s Population Grouping Methodology (similar to the Hierarchical Condition Category methods for risk-adjusting Medicare payments in the US^25^), calculated based on a regression model with total health care cost as the outcome predicted by a set of diagnostic variables and reported as the mean concurrent diagnosis-based risk score normalized to the full population of Ontario. The method has been discussed in detail elsewhere.^26,27,28^

### Study Period and Population

The unit of analysis was the referral. We included all new patient referrals occurring from April 1, 2018, to March 31, 2019, identified from OHIP claims containing consultation billing codes (eAppendix 1 in Supplement 1). Each referral included 1 year of follow-up from the date of initial consultation up to March 31, 2020. We removed cases in which the patient visited the same specialist in the prior 12 months. To focus the analyses on physicians who derived a significant share of their income from referrals, only surgical and medical specialties were included (eAppendix 2 in Supplement 1). Referrals for patients with no data on age, gender, or patient complexity and observations with missing data on specialists’ or referring physicians’ age or gender were excluded from the analyses.

The study population included specialist physicians who received a new patient referral during the study period and referring physicians requesting an initial consultation. Referring physicians must have been listed in the OHIP data when a consultation was billed for the specialist to receive remuneration.

### Defining a Referral and Calculating Revenue per Referral

We defined a referral using an episode-of-care approach. A referral began with the first specialist consultation and ended with the last encounter with the same specialist within 12 months. This captured follow-up services, such as reassessments, diagnostic tests, and surgical procedures. We determined the revenue of the referral from claims submitted to OHIP and based on fees listed in the OHIP Schedule of Benefits.

### Statistical Analysis

Our study was composed of 3 separate analyses. First, we used defined formulas to assess how much of the gender-based referral pay gap was attributable to differences in the number of referrals received vs revenue per referral. Second, we used difference-in-differences multivariable regression to estimate the association of gender-based differences with revenue per referral. Third, a case-control design was used to examine gender-based differences in physician-referral patterns. In each of the analyses, we considered referring physician and specialist gender, measured as a binary indicator.

#### Examining Gender-Based Referral Differences

Mean total payments (fees billed) for referrals to female and male specialists were compared to calculate the overall unadjusted, gender-based pay differential. This differential was the product of the revenue per referral and the total number of referrals. The separate contribution of each of these 2 factors to the pay differential was calculated by holding the other factor constant (ie, equal for both female and male physicians). eAppendix 3 in Supplement 1 gives details.

#### Estimating Gender-Based Differences in Referral Revenue

We examined the hypothesis that female and male specialists receive qualitatively different types of referrals by looking at the total fees paid for each referral. For this analysis, the log of the total fees was used, as payment data are typically skewed.

We used a difference-in-differences approach to investigate gender-based differences in the revenue per referral, which is a linear regression model controlling for the referring and specialist physician gender as well as their interaction term (eAppendix 4 in Supplement 1). This allowed us to account for some plausible explanations for a pay differential between female and male specialists. One possible source of gender-based difference occurs if female specialists typically receive lower-revenue referrals from male physicians than from female physicians, which may partly reflect differences in the type of referrals generated by female and male physicians to any gender (eg, if female physicians have more complex cases to refer than male physicians). Another possible source of gender-based difference occurs if female referring physicians tend to send lower-revenue referrals to female specialists rather than to male specialists, which may partly reflect differences in the practice styles between male and female specialists. The interaction term, or difference-in-differences parameter, controls for both of these possibilities, estimating the gender differences in referral revenue received due to differences in male physician referrals to female specialists vs male specialists. That is, it allowed us to account for variations in practice style and referral style that may have plausibly, at least partially, been for reasons other than any gender-based discrimination in referral decisions. Our approach differed from the standard approach to difference-in-differences analysis, as there was no before or after comparison. However, it was equivalent in that it aimed to exclude explanations for differences due to common shocks.

To account for other potential interpretations of the pay differential, we controlled for several other factors that may be associated with specialist billings. Variable choice was similar to factors used elsewhere in the literature.^3^ As differences in referral revenue may have been associated with systematic differences by specialist gender in the complexity of referred patients, we controlled for patient factors such as patient age, gender, and overall clinical complexity. We also controlled for specialist characteristics, including specialty, which may also have been associated with referral revenue. Other specialist characteristics included factors associated with the nature of services provided, such as tenure (years since graduation), academic status, practice setting (hospital, office, or mixed), and practice rurality, categorized into 4 groups based on the Rurality Index for Ontario score: large urban (0), urban (≥1 and <10), small urban (≥10 and <40), and rural (≥40). We also controlled for specialist factors associated with their availability, such as pregnancy or parental leaves, number of days worked (in which any services were billed), and part-time status (defined as having billings on <3 days per week).

All analyses were performed for the total and separately for the initial consultation and subsequent services. Subgroup analysis was done for surgical and medical specialists.

#### Identifying Referral Preferences

Following Zeltzer,^20^ we estimated gender-based differences in physicians’ referral patterns with a case-control design. Cases were defined as specialists chosen for the referral by a referring physician in the initial consultation, while controls were a random sample of specialists not chosen for referral by a referring physician from the same specialty and geographic health region (Local Health Integration Network) as the specialist that was chosen for referral. Specialists with missing data on geographic region were not included.

As in Zeltzer,^20^ because of the computational infeasibility of considering all possible combinations of specialists and referring physicians, choice-based sampling was used, wherein 2 randomly sampled, unchosen specialists were selected as controls to be compared with the specialist who was chosen for the referral. The final data included a record for each linked referring physician and specialist (1 case observation and ≤2 control observations). Cases that did not have any matched controls (ie, they were the only specialist of that type for the region) were removed from the analysis. Additionally, if both randomly selected controls were the same gender as the specialist who received the referral, that case was excluded since they could not be used to infer gender-based preferences.

Gender-based differences in the odds of receiving a referral were estimated using a conditional logit model. The outcome variable was a binary indicator equal to 1 if the physician referred to the specialist (case observation) and 0 if the specialist was not chosen (control observation). Explanatory variables in the model included gender of the specialist and whether the referring physician and specialist were the same gender, had similar tenure (within 5 years of experience), and performed most of their services at the same hospital.

Data were analyzed from April 2018 to March 2019 for initial consultations and from April 2018 to March 2020 when including a 12-month follow-up period. All analyses were conducted using Stata, version 15 (StataCorp LLC) and SAS, version 9.4 (SAS Institute Inc). Using 2-sided *t* tests, *P* < .05 was considered significant.

## Results

The data included 13 582 specialists, of whom 4890 (36.0%) were female (mean [SD] age, 45.6 [11.0] years) and 8692 (64.0%) were male (mean [SD] age, 51.8 [13.0] years); 32 824 referring physicians, of whom 13 512 (41.2%) were female (mean [SD] age, 46.3 [11.6] years) and 19 312 (58.8%) were male (mean [SD] age, 52.9 [13.5] years); 4 225 150 patients, of whom 2 344 788 (55.5%) were female and 1 880 362 (44.5%) were male; and 7 621 365 new referrals, of whom 2 115 256 (27.8%) were female specialists and 5 506 109 (72.2%) were male specialists. Compared with female specialists, male specialists were older, had more years of experience, and worked more days (Table 1). A higher percentage of female specialists had an academic status, worked part-time, had practices in large urban areas, and worked in hospital or office settings (vs a mixed setting) compared with male specialists. Male referring physicians were older and had more years of experience compared with female referring physicians. Total billings for new initial referrals in our study were estimated at $1.4 billion of $5.3 billion in total fee-for-service payments (26.4%) to those specialists for the 2018 to 2019 fiscal year.

**Table 1.** Demographic and Practice Characteristics of Specialists and Referring Physicians by Gender^a^

Characteristic	Female	Male	*P* value^b^
**Specialists (N = 13 582)**			
Total	4890 (36.0)	8692 (64.0)	NA
Age, mean (SD), y	45.6 (11.0)	51.8 (13.0)	<.001
Tenure, mean (SD), y	19.0 (11.3)	25.5 (13.5)	<.001
Time worked, mean (SD), d	167.1 (78.7)	200.5 (81.2)	<.001
Received payment from academic center	1616 (33.0)	2648 (30.5)	.002
Received PPLB payment	274 (5.6)	113 (1.3)	<.001
Part-time status	1925 (39.4)	2126 (24.5)	<.001
Rurality^c^			
Large urban	3538 (72.4)	5829 (67.1)	<.001
Urban	974 (19.9)	1955 (22.5)	.001
Small urban	298 (6.1)	751 (8.6)	<.001
Rural	60 (1.2)	110 (1.3)	.85
Missing	20 (0.4)	47 (0.5)	.29
Practice setting			
Hospital	2191 (44.8)	3688 (42.4)	.007
Office	1054 (21.6)	1605 (18.5)	<.001
Mixed	1645 (33.6)	3399 (39.1)	<.001
**Referring physicians (N = 32 824)**			
Total	13 512 (41.2)	19 312 (58.8)	NA
Age, mean (SD), y	46.3 (11.6)	52.9 (13.5)	<.001
Tenure, mean (SD), y	19.4 (11.9)	26.2 (14.1)	<.001
General practitioner specialty	6334 (49.9)	6857 (37.8)	<.001
Rurality^c^			
Large urban	8321 (61.6)	11 214 (58.1)	<.001
Urban	3040 (22.5)	4562 (23.6)	.02
Small urban	1462 (10.8)	2507 (13.0)	<.001
Rural	588 (4.4)	874 (4.5)	.45
Missing	101 (0.7)	155 (0.8)	.58

Abbreviations: NA, not applicable; PPLB, Pregnancy and Parental Leave Benefit Program.

^a^ Data are reported as number (percentage) of individuals unless otherwise indicated.

^b^ *P* values were derived from 2-sided *t* test.

^c^ Rurality categories were defined based on Rurality Index for Ontario scores (large urban [0], urban [≥1 and <10], small urban [≥10 and <40], and rural [≥40]).

### Unadjusted Analysis

Male specialists had a mean (SD) of 633 (666) new patient referrals and $350 ($474) fees billed per referral compared with 433 (515) and $316 ($393) for female specialists, respectively. This resulted in a total pay differential of $84 944.15, or a 38.3% unadjusted annual pay gap, for 1-year payments derived from new referrals (Table 2). Male specialists received referrals for a higher percentage of male patients (48.1% vs 36.8%; *P* < .001), older patients (53.7% vs 48.0%; *P* < .001), and patients with higher mean diagnostic complexity (Canadian Institute for Health Information population concurrent risk score: 6.7 vs 6.5; *P* < .001) compared with female specialists. Male specialists also received a higher percentage of referrals from male physicians (64.6% vs 56.8%; *P* < .001) than did female specialists.

**Table 2.** Number and Revenue of New Referrals by Specialist Gender^**a**^

	Female	Male
All referrals		
Total, No.	2 115 256	5 506 109
Mean (SD)	433 (515)	633 (666)
Revenue per referral, mean (SD), $	316 (393)	350 (474)
Revenue for all referrals, mean (SD), $	136 749 (140 590)	221 693 (223 595)
Medical specialty referrals		
Total, No.	1 321 824	2 796 051
Mean (SD)	393 (522)	562 (672)
Revenue per referral, mean (SD), $	329 (394)	361 (392)
Revenue for all referrals, mean (SD), $	129 072 (133 172)	202 874 (209 632)
Surgical specialty referrals		
Total, No.	793 432	2 710 058
Mean (SD)	520 (487)	729 (647)
Revenue per referral, mean (SD), $	295 (391)	338 (546)
Revenue for all referrals, mean (SD), $	153 691 (154 414)	246 906 (238 753)
Referrals that included subsequent encounters, No./total No. (%)	1 021 008/2 115 256 (48.3)	2 730 896/5 506 109 (49.6)
Revenue per initial consultation, mean (SD), $	176 (130)	186 (194)
Revenue for all initial consultations, mean (SD), $	75 933 (87 600)	117 876 (134 806)
With any subsequent encounters, mean (SD), No.	217 (245)	324 (341)
Revenue per referral for subsequent encounters, mean (SD), $	292 (481)	330 (564)
Revenue for all subsequent encounters, mean (SD), $	63 235 (74 773)	107 006 (125 360)

^a^ All *P* results were statistically significant at *P* <.001, derived from 2-sided *t* tests.

Of the male to female differential in total annual payments from new referrals, 82.8% was due to differences in the number of referrals received, while 17.2% was due to a difference in the revenue per referral (Table 3). The proportion of the differential accounted for by a difference in the revenue per referral was larger for surgical specialties (24.0%) compared with medical specialties (17.3%). Stratifying referrals into the initial consult vs all subsequent encounters, we found almost no difference between medical and surgical specialties in the portion of the differential explained by revenue per referral for initial consultations (19.0% vs 17.8%, respectively). However, the portion accounted for by revenue per referral attributable to subsequent encounters was larger for surgical specialties (26.6%) compared with medical specialties (8.6%). The distribution of revenue from referrals by specialist gender is shown in eFigure 2 in Supplement 1.

**Table 3.** Referral Pay Differential Attributed to Number vs Revenue From Referrals

Visit, specialty	Referral pay differential, $^a^	Referrals per specialist, mean (SD), No.		Revenue from referrals to specialists, mean (SD), $		Pay differential, %	
		Female	Male	Female	Male	From No. of referrals^b^	From revenue from referrals^c^
**Initial consultation and subsequent encounters**							
All	−84 944.15	433 (515)	633 (666)	316.13 (393.30)	349.97 (474.18)	82.8	17.2
Surgical	−93 215.05	520 (487)	729 (647)	295.40 (390.97)	338.46 (546.10)	76.0	24.0
Medical	−73 802.51	393 (522)	562 (672)	328.58 (394.18)	361.12 (391.75)	82.7	17.3
**Initial consultation**							
All	−41 943.12	433 (515)	633 (666)	175.54 (130.12)	186.08 (194.08)	89.1	10.9
Surgical	−38 162.13	520 (487)	729 (647)	136.90 (144.97)	149.95 (215.66)	82.2	17.8
Medical	−46 145.23	393 (522)	562 (672)	198.73 (114.21)	221.10 (163.10)	81.0	19.0
**Subsequent encounters**							
All	−43 771.37	217 (245)	324 (341)	291.27 (481.25)	330.43 (564.14)	80.6	19.4
Surgical	−56 543.08	269 (272)	380 (368)	318.73 (461.46)	374.57 (649.03)	73.4	26.6
Medical	−27 799.89	193 (228)	282 (313)	273.98 (492.52)	286.40 (460.01)	91.4	8.6

^a^ The difference in mean revenue from referrals to female vs male specialists.

^b^ Calculated as *p_m_(n_F_ − n_m_) / y_F_ – y_m_*, where *p* indicates revenue per referral, *m* indicates male specialists, *n* indicates number of referrals, *F* indicates female specialists, and *y* indicates total revenue.

^c^ Calculated as *n_F_(p_F_  p_m_) / y_F_ – y_m_*, where *p* indicates revenue per referral, *m* indicates male specialists, *n* indicates number of referrals, *F* indicates female specialists, and *y* indicates total revenue.

### Revenue per Referral

The difference-in-differences estimate of the unadjusted pay differential per referral between male and female specialists was −3.99% (95% CI, −4.24% to −3.74%) (Table 4); a male physician referring to a female specialist, compared with a referral to a male specialist, was associated with a 4.0% decrease in referral revenue. Adjusting for physician and patient characteristics, this difference was −4.68% (95% CI, −4.89% to −4.46%). The adjusted estimates were lower for initial consultations (−2.22%; 95% CI, −2.36% to −2.09%) and for subsequent services (−3.49%; 95% CI, −3.97% to −3.01%). Estimates were lower for medical specialties and higher for surgical specialties compared with overall results (eTables 1 and 2 in Supplement 1).

**Table 4.** Regression of Revenue per Referral as a Function of Specialist and Referring Physician Gender^a^

	Unadjusted analysis, percentage points (95% CI)			Adjusted analysis, percentage points (95% CI)^b^			
	Total (N = 7 621 365)	Initial consultation (n = 7 621 365)	Subsequent encounters (n = 3 751 904)	Total (N = 7 451 390)	Initial consultation (n = 7 451 390)	Subsequent encounters (n = 3 671 113)
Female specialist^c^	−1.33 (−1.52 to −1.14)	0.54 (0.41 to 0.68)	−1.79 (−2.18 to −1.41)	−0.9 (−1.08 to 0.74)	−2.70 (−2.80 to −2.59)	−1.93 (−2.31 to −1.54)
Male referring physician^d^	4.75 (4.61 to 4.89)	3.32 (3.22 to 3.42)	4.68 (4.40 to 4.97)	3.37 (3.24 to 3.50)	3.58 (3.49 to 3.66)	0.80 (0.52 to 1.08)
Female specialist × male referring physician^e^	−3.99 (−4.24 to −3.74)	−2.02 (−0.38 to −0.03)	−3.70 (−4.21 to −3.19)	−4.68 (−4.89 to −4.46)	−2.22 (−2.36 to −2.09)	−3.49 (−3.97 to −3.01)
Adjusted *R*^2^	0.001	0.001	0.001	0.234	0.375	0.118

^a^ Regression of revenue per referral was the log of total payments.

^b^ Controlled for physician specialty; specialist tenure, tenure squared, and academic status; pregnancy or parental leave status; part-time status; number of working days; practice rurality; practice setting; referring physician tenure and tenure squared; and patient age, gender, and complexity.

^c^ Reference is male.

^d^ Reference is female.

^e^ Difference-in-differences estimate.

### Propensity to Refer

In the case-control conditional logit analysis, referring physicians had higher odds of referring to specialists of the same gender (odds ratio [OR], 1.07; 95% CI, 1.06-1.07). However, some of this difference was found to be attributable to other factors. Although the likelihood to refer to specialists with the same gender remained significant (OR, 1.04; 95% CI, 1.03-1.04), adjusted analysis also revealed higher odds of referring to male specialists (OR, 1.10; 95% CI, 1.09-1.11), specialists with similar years of experience (OR, 1.12; 95% CI, 1.12-1.13), and specialists practicing at the same hospital (OR, 8.44; 95% CI, 8.36-8.53). Results were similar for medical and surgical specialties except for differences in odds of referring to male specialists (medical: OR, 1.12 [95% CI, 1.11-1.13]; surgical: OR, 1.06 [95% CI, 1.05-1.07]) (Table 5). Subgroup analysis by female and male referring physicians is shown in eTable 3 in Supplement 1.

**Table 5.** Odds of Receiving Referral Based on Specialist Gender and Concordance With Key Referring Physician Characteristics in Case-Control Conditional Logit Regression Analysis^a^

Referral characteristic	Odds ratio (95% CI)		
	All specialties	Medical specialties	Surgical specialties
Same gender			
No	1 [Reference]	1 [Reference]	1 [Reference]
Yes	1.04 (1.03-1.04)	1.03 (1.02-1.04)	1.04 (1.03-1.05)
Gender of specialist			
Female	1 [Reference]	1 [Reference]	1 [Reference]
Male	1.10 (1.09-1.11)	1.12 (1.11-1.13)	1.06 (1.05-1.07)
Similar tenure			
No	1 [Reference]	1 [Reference]	1 [Reference]
Yes	1.12 (1.12-1.13)	1.13 (1.12-1.13)	1.12 (1.11-1.13)
Same hospital			
No	1 [Reference]	1 [Reference]	1 [Reference]
Yes	8.44 (8.36-8.53)	8.37 (8.26-8.47)	8.54 (8.42-8.67)
Observations, No.			
Total	3 377 613	1 805 129	1 572 484
Cases	1 222 532	664 384	558 148
Controls	2 155 081	1 140 745	1 014 336

^a^ All analyses were controlled for specialist gender if the referring physician and specialist were the same gender, had similar tenure (≥5 or <5 years of experience), and performed most of their services at the same hospital. *P* < .001 for all estimates.

## Discussion

This cross-sectional study found that 82.8% of the unadjusted, referral-generated gender pay gap among specialists in Ontario was due to the difference in the number of referrals, while the remaining 17.2% was due to the difference in revenue per referral. Thus, understanding differences in the number of referrals vs the referral revenue may be more consequential for closing the pay gap. Generalizability of results may depend on institutional features of jurisdictions. However, systematic differences depend on training and culture, which have significant overlap across high-income countries.

We documented a difference of 4.7% in revenue per referral between male and female specialists after controlling for physician and patient characteristics. That is, in the absence of gender-based difference, female specialists would earn 4.7% more per referral. These findings are consistent with prior research suggesting that part of the pay gap in referrals may be attributed to male specialists having more lucrative referrals^2^ and more procedural referrals.^1^ Differences in revenue per referral may be driven by several mechanisms, including systematic differences in the complexity of referred patients or gender-based differences in billing or practice style. It is not clear whether higher referral revenue for male vs female specialists in the 1-year follow-up period was beneficial and appropriate or reflected overuse. While effect sizes were small, the cumulative consequences over time for lifetime earnings may be larger, as fewer or less complex referrals to female specialists could create a cycle, with lower skill accumulation and fewer opportunities to perform procedures.^22^

We also found that physicians were more likely to refer to the same gender. Dossa et al^1^ found that female physicians had 1.6% greater odds of referring to female surgeons, while male physicians had 32% greater odds of referring to male surgeons. As in our study, there was a higher proportion of male referring physicians and physicians had higher odds of referring to specialists of the same gender; increased participation of female referring physicians may help minimize the gender gap in earnings.^20^ However, we found physicians of both genders disproportionately referred to male specialists, affecting the total number of referrals and hence total remuneration of each specialist. Furthermore, a study using data from Ontario found that surgical specialties with the highest proportion of women had the greatest referral inequities, and disparities persisted over time.^1^ Therefore, equal representation of women in the medical profession may be insufficient to eliminate gender-based referral bias.

Another suggestion to eliminate gender-based referral bias is to create a gender-blind, centralized, and transparent referral system.^15^ Providing referring physicians with specialists’ individual wait times to ensure an equitable distribution of patients across specialists may also be an advantage for female physicians who are suspected to have shorter waiting lists compared with male physicians due to referral biases.^15^ This approach may limit freedom of choice with respect to referrals, but this disadvantage may be offset if the wait times are shorter.^29^ Centralized referral systems designed to improve timely access to procedures have only recently been implemented in Ontario for hip and knee replacement and anorectal surgeries,^30,31^ and there is still a dearth of evidence as to whether these systems reduce gender-based biases in referrals. However, a recent qualitative study suggested such systems may be gaining acceptability in the current environment, as solutions are needed to address the surgical backlogs precipitated by SARS-CoV-2 infection.^32^

### Limitations

This study has some important limitations. First, we examined fee-for-service billings for referrals for new patients only, which covered only about one-third of specialists’ total billings. Second, we lacked data on consultant subspecialty, which may have affected a physician’s decision to refer patients to a particular specialist. Third, several factors that prior studies have found to be associated with referral decisions were not controlled for in this study (eg, fear of malpractice, belief systems, and psychosocial orientation).^33,34^ Fourth, we did not have information on whether there was any gender-based difference in the likelihood of a specialist to refuse referrals. Fifth, while our difference-in-differences approach reduced the number of potential interpretations of gender-based differences in referrals, results could not conclusively be interpreted as deliberate bias or discrimination due to the potential for unobserved confounders. Furthermore, as our difference-in-differences design was not based on before and after periods, we could not test for a common trend in the before period, meaning causal interpretation could not be established. Additionally, nonbinary gender and other relevant physician characteristics that may have intersected with gender and been associated with referral biases, such as transgender status, gender orientation, and race and ethnicity, were not captured in the data.

## Conclusions

This cross-sectional study found gender-based differences in the propensity to receive a physician referral and revenue from referrals received. Female specialists earned less in revenue per referral than did males, a male physician referring to a female specialist was associated with a lower referral revenue, and referring physicians had higher odds of referring to specialists of the same gender. These findings may help explain 1 dimension of the gender pay gap in medicine. A centralized referral system has been suggested as a potential solution to address this gap, but more research is needed to explore the effectiveness of this and other policies.

## Notes

Supplement 1. **eFigure 1.** STROBE Flow Diagram **eAppendix 1.** Background on Consultation Referrals in Ontario, Canada **eAppendix 2.** List of Medical and Surgical Consultation Codes in Ontario Used in This Study **eAppendix 3.** Calculating Proportions of the Annual Referral Income Differential Explained by Differences in the Number and Value of Referrals **eAppendix 4.** Calculating the Impact of Gender-Based Differences Upon Specialist Pay **eTable 1.** OLS Regression of the Value per Referral (Log of Total Payments) as a Function of Specialist and Referring Physician Gender (95% CI), Unadjusted, by Medical vs Surgical Specialties **eTable 2.** OLS Regression of the Value per Referral (Log of Total Payments) as a Function of Specialist and Referring Physician Gender (95% CI), Adjusted, by Medical vs Surgical Specialties **eTable 3.** Odds of Receiving Referral Based on Specialist Gender and Concordance With Key Referring Physician Characteristics, in Case-Control Conditional Logit Regression Analysis (95% CI), by Referring Physician Gender (95% CI) **eFigure 2.** Distribution of Referral Value by Specialist Gender

Supplement 2.
Data Sharing Statement
