# Supplementary material for: Referring and Specialist Physician Gender and Specialist Billing
Source: JAMA Netw Open. 2023 Aug 25;6(8):e2328347. doi: 10.1001/jamanetworkopen.2023.28347 (PMC10457710; doi:10.1001/jamanetworkopen.2023.28347)
Supplement: Supplement 1. — eFigure 1. STROBE Flow Diagram eAppendix 1. Background on Consultation Referrals in Ontario, Canada eAppendix 2. List of Medical and Surgical Consultation Codes in Ontario Used in This Study eAppendix 3. Calculating Proportions of the Annual Referral Income Differential Explained by Differences in the Number and Value of Referrals eAppendix 4. Calculating the Impact of Gender-Based Differences Upon Specialist Pay eTable 1. OLS Regression of the Value per Referral (Log of Total Payments) as a Function of Specialist and Referring Physician Gender (95% CI), Unadjusted, by Medical vs Surgical Specialties eTable 2. OLS Regression of the Value per Referral (Log of Total Payments) as a Function of Specialist and Referring Physician Gender (95% CI), Adjusted, by Medical vs Surgical Specialties eTable 3. Odds of Receiving Referral Based on Specialist Gender and Concordance With Key Referring Physician Characteristics, in Case-Control Conditional Logit Regression Analysis (95% CI), by Referring Physician Gender (95% CI) eFigure 2. Distribution of Referral Value by Specialist Gender [file jamanetwopen-e2328347-s001.pdf]

Supplemental Online Content

Chami N, Weir S, Shaikh SA, et al. Referring and specialist physician gender and specialist billing. *JAMA Netw Open*. 2023;6(8):e2328347.
doi:10.1001/jamanetworkopen.2023.28347

eFigure 1. STROBE Flow Diagram
eAppendix 1. Background on Consultation Referrals in Ontario, Canada
eAppendix 2. List of Medical and Surgical Consultation Codes in Ontario Used in This Study
eAppendix 3. Calculating Proportions of the Annual Referral Income Differential Explained by Differences in the Number and Value of Referrals
eAppendix 4. Calculating the Impact of Gender-Based Differences Upon Specialist Pay
eTable 1. OLS Regression of the Value per Referral (Log of Total Payments) as a Function of Specialist and Referring Physician Gender (95% CI), Unadjusted, by Medical vs Surgical Specialties
eTable 2. OLS Regression of the Value per Referral (Log of Total Payments) as a Function of Specialist and Referring Physician Gender (95% CI), Adjusted, by Medical vs Surgical Specialties
eTable 3. Odds of Receiving Referral Based on Specialist Gender and Concordance With Key Referring Physician Characteristics, in Case-Control Conditional Logit Regression Analysis (95% CI), by Referring Physician Gender (95% CI)
eFigure 2. Distribution of Referral Value by Specialist Gender

This supplemental material has been provided by the authors to give readers additional information about their work.

© 2023 Chami N et al. *JAMA Network Open*.

eFigure 1. STROBE Flow diagram

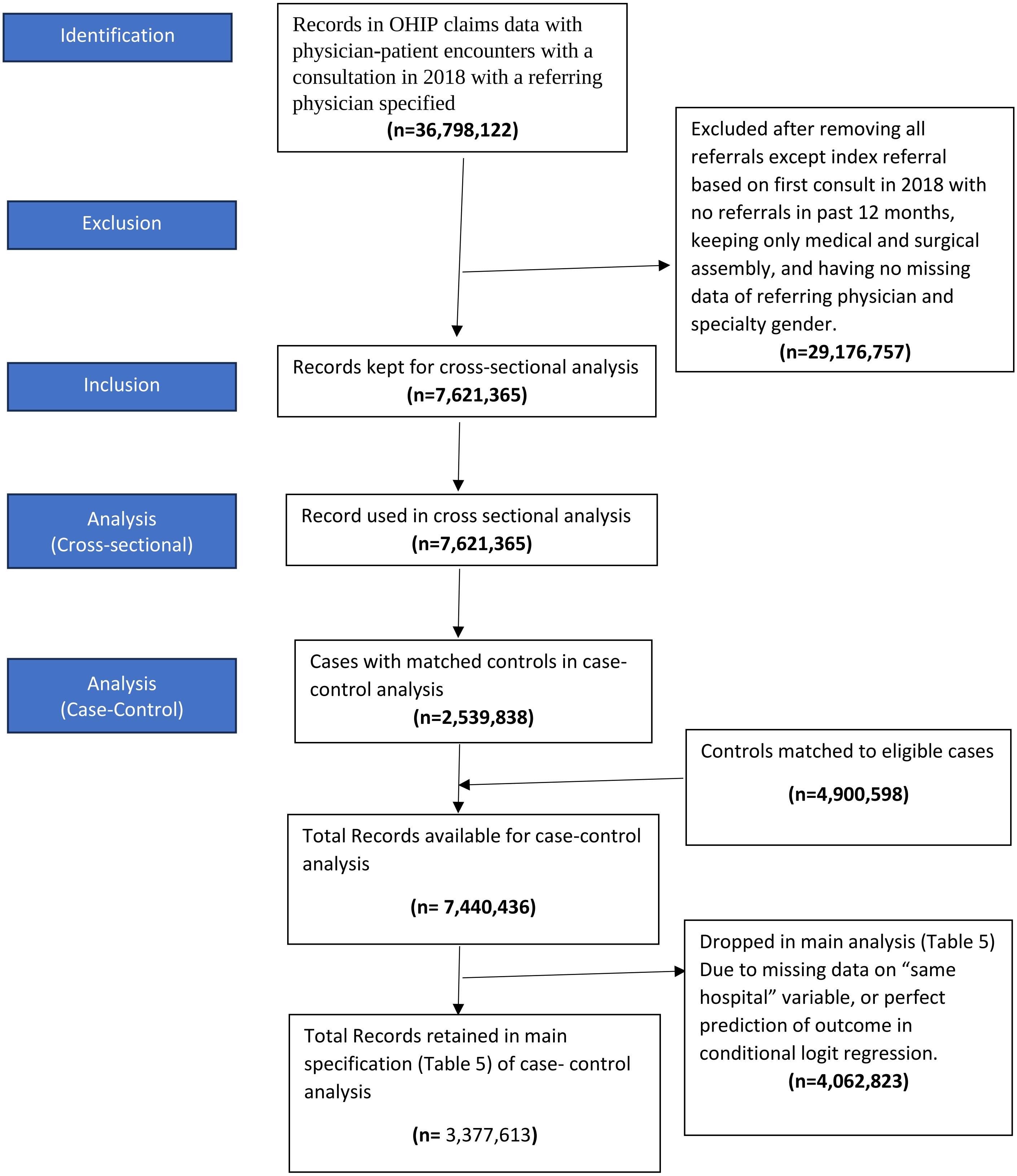

© 2023 Chami N et al. *JAMA Network Open*.

eAppendix 1. Background on Consultation Referrals in Ontario, Canada

In Ontario, consultation referrals are generated by a written request from a physician, usually a family physician or nurse practitioner, seeking the professional opinion of a consultant physician, usually a specialist, who is qualified to provide advice for patients with complex, serious, or uncertain cases.[1] The written request typically specifies the services required for the referred patient and includes the reason the patient is being referred, relevant patient clinical information, medical history, physical findings, and laboratory test reports, and urgency of the consultation. The consultant physician will then acknowledge the referral within 14 days of the request, indicating if they accept or decline the referral. If a referral is accepted, an appointment date is set by the consulting physician, and if it is not, a reason for declining the referral is provided.[2]

In order to receive payment for a consultation referral, the consultant physician must bill the specific consultation fee code for their specialty and provide the billing number of the referring physician. As of October 1, 2019, payment rules precluded specialists from billing a consultation for the same patient for the same diagnosis within 24 months of the previous consultation if performed in an office setting, and within 12 months if the patient was admitted to hospital or seen in the Emergency Department (ED).[3]

References

1. Ontario Health Insurance Plan (OHIP) Schedule of Benefits, Physician Services Under the Health Insurance Act, July 2, 2021. Available at https://www.health.gov.on.ca/en/pro/programs/ohip/sob/physserv/sob_master.pdf Accessed 15 June 2021.

2. The College of Physicians and Surgeons of Ontario (CPSO), Transitions in Care, September 2019. Available at https://www.cpso.on.ca/Physicians/Policies-Guidance/Policies/Transitions-in-Care Accessed 15 June 2021.

3. Ontario Health Insurance Plan InfoBulletin 4726, August 22, 2019. Available at https://www.health.gov.on.ca/en/pro/programs/ohip/bulletins/4000/bul4726.aspx Accessed 15 June 2021.

© 2023 Chami N et al. *JAMA Network Open*.

eAppendix 2. List of Medical and Surgical Consultation Codes in Ontario Used in This Study

Medical Specialties listed under the Ontario Medical Association Assembly definition

Specialty	Consultation billing code*	Description of code
Dermatology	A025	Dermatology - consultation
Internal Medicine	A135	Internal Medicine - consultation
Endocrinology	A155	Endocrinology - consultation
Nephrology	A165	Nephrology - consultation
Neurology	A185	Neurology - consultation
Psychiatry	A195	Psychiatry - consultation
Genetics	A225	Genetics - consultation
Paediatrics	A265	Paediatrics - consultation
Physical Medicine	A315	Physical Medicine and Rehabilitation - consultation
Therapeutic Radiology	A335	Diagnostic Radiology - consultation
Gastroenterology	A415	Gastroenterology - consultation
Medical Oncology	A445	Medical Oncology - consultation
Infectious Disease	A465	Infectious Diseases - consultation
Respiratory Diseases	A475	Respiratory Disease - consultation
Rheumatology	A485	Rheumatology - consultation
Cardiology	A605	Cardiology - consultation
Haematology	A615	Haematology - consultation
Clinical Immunology	A625	Clinical Immunology - consultation

Surgical Specialties listed under the Ontario Medical Association Assembly definition

Specialty	Consultation billing code*	Description of code
All surgeons	A935	Special surgical consultation
Cardiac surgery	A095	Consultation
General surgery	A035	Consultation
Thoracic surgery	A645	Consultation
Gynecology	A205	Consultation
Ophthalmology	A235	Consultation
	A251	Special ophthalmologic assessment
Orthopedic surgery	A065	Consultation
Otolaryngology	A245	Consultation
Plastic surgery	A085	Consultation
Urology	A355	Consultation
Vascular surgery	A175	Consultation
Neurosurgery	A045	Consultation

*Anaesthesia and surgical assistant codes were not included.

© 2023 Chami N et al. *JAMA Network Open*.

eAppendix 3. Calculating Proportions of the Annual Referral Income Differential Explained by Differences in the Number and Value of Referrals

We decomposed the proportion of the annual referral income differential that can be explained by differences in the number of referrals (holding value per referral constant) and the proportion that can be explained by differences in the value per referral (holding the number of referrals constant) by starting with the equation for the pay differential in referral income:

$$y_F - y_m = p_F \times n_F - p_m \times n_m$$

where $y_F \ and \ y_m$ are the average total income (payments) derived from referrals for female and male specialists; $p_F \ and \ p_m$ are the average value for a referral received by female and male specialists; and $n_F \ and \ n_m$ are the average number of referrals to female and male specialists.

The income differential owing to the value of referrals was acquired by setting the number of referrals equal for male and female specialists. Holding the number of referrals constant, $n_F = n_m$, equation (1) becomes:

$$y_F - y_m = p_F \times n_F - p_m \times n_F = n_F(p_F - p_m)$$

Therefore, the proportion that can be explained by differences in the value per referral is:

$$\frac{n_F(p_F - p_m)}{y_F - y_m}$$

The income differential owing to the number of referrals was acquired by setting the average value per referral equal for male and female specialists. Holding the value of referrals constant, $p_F = p_m$, equation (1) becomes:

$$y_F - y_m = p_m \times n_F - p_m \times n_m = p_m(n_F - n_m)$$

Therefore, the proportion that can be explained by differences in the number of referrals is:

$$\frac{p_m(n_F - n_m)}{y_F - y_m}$$

© 2023 Chami N et al. *JAMA Network Open*.

eAppendix 4. Calculating the Impact of Gender-Based Differences Upon Specialist Pay

To observe the association of physician gender with referral payments, the log of total payments per referral was estimated as a function of gender of referring physician and specialist physician, and the interaction of referring and specialist physician gender as follows:

$$y_i = \alpha + \beta S_i + \gamma R_i + \delta S_i R_i + \varepsilon_i$$

where y_i is the value of services associated with referral i, $S_i = 1$ if the specialist physician is female and 0 if male, $R_i = 1$ if the referring physician is male and 0 if female, and ε_i is the error term (which in the analysis will be divided into observable and unobservable characteristics, but this distinction is suppressed here for simplicity of exposition). α is a constant representing the value of the referral if referring physician is female and specialist is male, β represents the difference in value of the referral if the specialist physician is female, and γ represents the difference in value of the referral if the referring physician is male. δ represents the difference in value of the referral if the specialist is female and referring physician is male.

This generates the following matrix of the value of services billed for referral *i* that are associated with the gender of referring and specialist physicians.

		Referring Physician	
		$R_i = 0$ (female)	$R_i = 1$ (male)
Specialist Physician	$S_i = 0$ (male)	α	$\alpha + \gamma$
	$S_i = 1$ (female)	$\alpha + \beta$	$\alpha + \beta + \gamma + \delta$

The impact upon payments that is associated with the combination of female referring physician and male specialist is captured by the estimate for the constant, α. The impact of gender of referring physician being female and gender of specialist physician being female may be obtained by summing the estimates for the constant with the coefficient for female specialist, $\alpha + \beta$. The impact upon payments for a male specialist who receives a referral from a male referring physician is captured by adding the coefficient associated with female referring physician to the constant, $\alpha + \gamma$. To estimate the impact of the combination of male referring physician and female specialist, it is necessary to sum the constant, the coefficients for female

© 2023 Chami N et al. *JAMA Network Open*.

specialist and male referring physician and the coefficient on the interaction term, $\alpha + \beta + \gamma + \delta$.

One possible source of gender-based difference occurs if female specialists typically receive referrals of lesser value from male physicians than from female physicians:

$$E[y_i|S_i = 1, R_i = 1] - E[y_i|S_i = 1, R_i = 0] = \gamma + \delta < 0$$

However, this may partly reflect differences in the type of referrals generated by male and female physicians to any gender. For example, female physicians may refer higher value (e.g., more complex) cases than do male physicians, on average, regardless of who they refer to (i.e., $\gamma < 0$).

Another possible source of gender-based difference occurs if female referring physicians tend to send referrals of lesser value to female specialists rather than to male specialists:

$$E[y_i|S_i = 1, R_i = 0] - E[y_i|S_i = 0, R_i = 0] = \beta < 0$$

However, this may partly reflect differences in the type of practice between male and female specialists. For example, for any number of reasons other than discrimination, female physicians may bill less than male physicians bill for the same services, or owing differences in subspecialisation, may accept referrals for patients that are less complex.

We can rule out some gender-based differences in practice patterns of referring and specialist physicians using a difference-in-differences approach that compares how female vs male referring physicians treat female vs male specialists as follows:

$$\{E[y_i|S_i = 1, R_i = 1] - E[y_i|S_i = 1, R_i = 0]\} - \{E[y_i|S_i = 0, R_i = 1] - E[y_i|S_i = 0, R_i = 0]\} = \delta$$

The first part of the equation above compares payments for female specialists from female vs male referring physicians. The second part compares payments for male specialists from female vs male referring physicians. By taking the difference in differences, we focus attention on the differences in the referral value of payment received by male and female specialists due to differences in male physician referrals to female specialists.

Difference-in-differences (DiD) study designs are commonly used in healthcare research to address issues of confounding in observational studies when estimating the impact of an intervention.[1] A DiD design requires definition of a group that receives an intervention or "treatment" for which we seek to estimate the impact of the treatment, or "treatment effect" based on the difference-in-differences parameter, δ. For our analysis, the treatment effect represents the impact of referral by a male physician compared to a female physician on referral value, or the gender-based difference in referral value. Estimating the treatment effect is complicated by the fact that, for the treatment group - that is referrals made by male physicians - we cannot observe what the value of referrals would be, if they were made by female physicians. This is referred to as a "counterfactual" outcome. Rather, we construct an estimate for this

© 2023 Chami N et al. *JAMA Network Open*.

counterfactual outcome using an adequate comparison group under a set of assumptions. In a DiD design the counterfactual is constructed using the difference between two subgroups which do not receive the treatment. In our analysis, this is

$$E[y_i|S_i = 0, R_i = 0] - E[y_i|S_i = 1, R_i = 0],$$

which is the difference between expected referral value between male and female specialists who are referred by female physicians. The DiD design requires an assumption, that this quantity, which is observed in the data, can be used as an estimate of the counterfactual difference in referral values for specialists who received referrals by males, if they were in fact referred by female physicians instead. As the latter quantity cannot be observed, this core identifying assumption of the DiD design is not testable. This assumption is often referred to as a "common trend" assumption because in the typical implementation of DiD, this difference is usually based on two time periods (i.e., before and after a policy) in the comparison group. However, it is not necessary that these groups be time-periods so long as the basic identifying assumptions of DiD hold, and applications exist in the literature using gender subgroups in place of time-periods.[2]

For an in-depth technical discussion of assumptions underpinning difference-in-differences study designs, please see Zeldow and Hatfield.[3]

References

1. Dimick JB, Ryan AM. Methods for Evaluating Changes in Health Care Policy: The Difference-in-Differences Approach. *JAMA.* 2014;312(22):2401–2402. doi:10.1001/jama.2014.16153

2. Card D, Domnisoru C, Sanders SG, Taylor L, Udalova V. The Impact of Female Teachers on Female Students' Lifetime Well-Being. National Bureau of Economic Research; 2022 Sep 5.

3. Zeldow B, Hatfield L. Difference-in-Differences. 2019. Accessed May 15th, 2023. https://diff.healthpolicydatascience.org/

© 2023 Chami N et al. *JAMA Network Open*.

eTable 1. OLS Regression of the Value per Referral (Log of Total Payments) as a Function of Specialist and Referring Physician Gender (95% CI), Unadjusted, by Medical vs Surgical Specialties

	All Referrals		Index Consultations		Subsequent Encounters	
**Dependent variable = log of total fees paid**	**Medical**	**Surgical**	**Medical**	**Surgical**	**Medical**	**Surgical**
	(1)	(2)	(3)	(4)	(5)	(6)
**Gender of specialist (base=male)**						
Female	-0.0925	0.0463	-0.0752	0.0208	-0.0525	0.0268
	(-0.0948, -0.0902)	(0.0431, 0.0495)	(-0.0768, -0.0735)	(0.0190, 0.0226)	(-0.0571, -0.0479)	(0.0202, 0.0333)
**Gender of referring MD (base=female)**						
Male	0.0816	0.0340	0.0679	0.0316	0.0503	0.0456
	(0.0799, 0.0833)	(0.0318, 0.0363)	(0.0667, 0.0692)	(0.0303, 0.0330)	(0.0467, 0.0538)	(0.0411, 0.0501)
**Female specialist*Male referring MD**	-0.00835	-0.1290	-0.00501	-0.0411	-0.0012	-0.0981
	(-0.0113, -0.0054)	(-0.1330, -0.1250)	(-0.0071, -0.0029)	(-0.0434, -0.0387)	(-0.0073, 0.0049)	(-0.1070, -0.0894)
constant	5.564	5.2845	5.2044	4.7532	5.0807	5.0452
	(5.5626, 5.5653)	(5.2827, 5.2862)	(5.2034, 5.2053)	(4.7522, 4.7543)	(5.0779, 5.0835)	(5.0416, 5.0488)
N	4,117,875	3,503,490	4,117,875	3,503,490	1,993,460	1,758,444
adj. R-sq	0.008	0.001	0.010	0.001	0.001	0.000

© 2023 Chami N et al. *JAMA Network Open*.

eTable 2. OLS Regression of the Value per Referral (Log of Total Payments) as a Function of Specialist and Referring Physician Gender (95% CI), Adjusted, by Medical vs Surgical Specialties

	All Referrals		Index Consultations		Subsequent Encounters	
**Dependent variable = log of total fees paid**	**Medical**	**Surgical**	**Medical**	**Surgical**	**Medical**	**Surgical**
	(1)	(2)	(3)	(4)	(5)	(6)
**Gender of specialist (base=male)**						
Female	-0.0076 (-0.0095, -0.0058)	-0.0184 (-0.0218, -0.0150)	-0.0201 (-0.0213, -0.0188)	-0.0387 (-0.0407, -0.0368)	-0.0164 (-0.0207, -0.0121)	-0.0356 (-0.0416, -0.0275)
**Gender of referring MD (base=female)**						
Male	0.0225 (0.0210, 0.0239)	0.0442 (0.0420, 0.0463)	0.0319 (0.0309, 0.0329)	0.0390 (0.0376, 0.0403)	0.0081 (0.0048, 0.0114)	0.0066 (0.0021, 0.0112)
**Female specialist*Male referring MD**	-0.0270 (-0.0294, -0.0246)	-0.0662 (-0.0703, -0.0622)	-0.0207 (-0.0223, -0.0191)	-0.0226 (-0.0250, -0.0203)	-0.0116 (-0.0171, -0.0061)	-0.0571 (-0.0658, -0.0485)
constant	5.4387 (5.4340, 5.4433)	4.7647 (4.7570, 4.7724)	5.3031 (5.3000, 5.3062)	4.7560 (4.7512, 4.7608)	5.0105 (4.9995, 5.0215)	4.1968 (4.1807, 4.2130)
N	4,029,701	3,421,689	4,029,701	3,421,689	1,953,768	1,717,345
adj. R-sq	0.338	0.116	0.434	0.073	0.191	0.073

Adjusted analysis controlled for specialist tenure, specialist tenure squared, referring physician tenure, referring physician tenure squared, patient age, gender, and complexity, specialist academic status, pregnancy/parental leave status, part-time status, number of working days, practice rurality, and practice setting.

© 2023 Chami N et al. *JAMA Network Open*.

eTable 3. Odds of Receiving Referral Based on Specialist Gender and Concordance With Key Referring Physician Characteristics, in Case-Control Conditional Logit Regression Analysis (95% CI)[a], by Referring Physician Gender (95% CI)

	Female Referring Physicians			Male Referring Physicians		
	**(1)**	**(2)**	**(3)**	**(5)**	**(6)**	**(7)**
**Gender of specialist (base=female)**						
Male	1.048 (1.043, 1.054)	1.057 (1.051, 1.062)	1.061 (1.052, 1.071)	1.164 (1.159, 1.170)	1.166 (1.160, 1.171)	1.138 (1.129, 1.146)
**Similar tenure (base=no)**						
Yes		1.144 (1.138, 1.150)	1.127 (1.117, 1.137)		1.141 (1.136, 1.147)	1.123 (1.115, 1.131)
**Same hospital (base=no)**						
Yes			7.966 (7.841, 8.092)			8.732 (8.627, 8.838)
Number of Referrals	3,187,928	3,187,928	1,250,886	4,252,508	4,252,508	2,126,727

[a] All analyses were adjusted for specialty, tenure, practice setting, academic status, part-time status, pregnancy/parental leaves, and practice rurality.

[b] P-values of all estimates are <0.001.

© 2023 Chami N et al. *JAMA Network Open*.

eFigure 2. Distribution of Referral Value by Specialist Gender

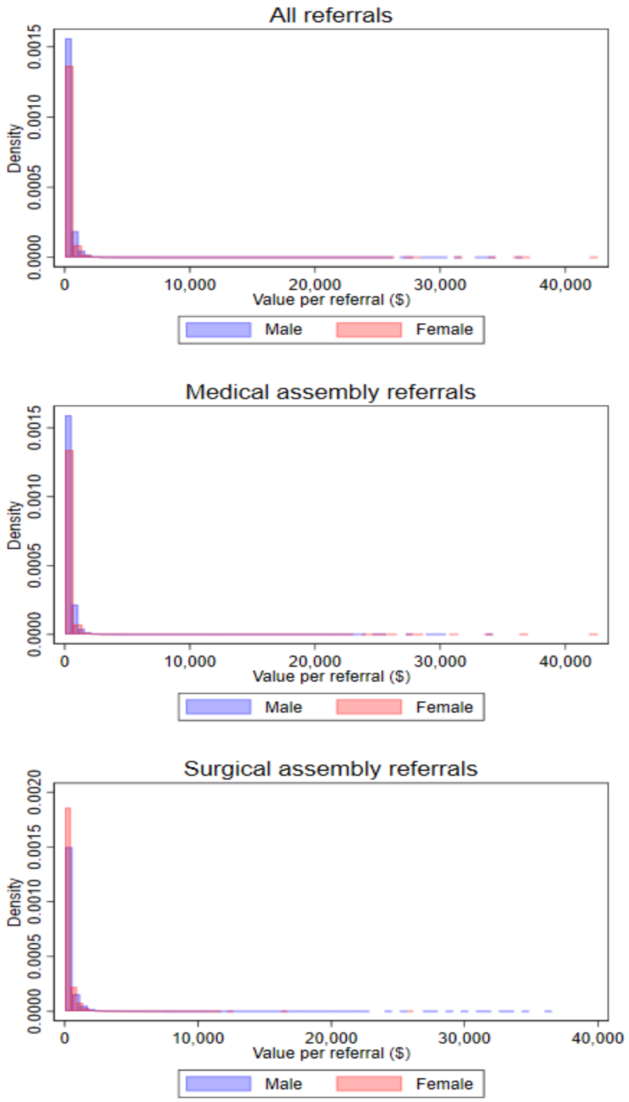

© 2023 Chami N et al. *JAMA Network Open*.
