# Supplementary material for: Referring and Specialist Physician Gender and Specialist Billing
Source: JAMA Netw Open. 2023 Aug 25;6(8):e2328347. doi: 10.1001/jamanetworkopen.2023.28347 (PMC10457710; doi:10.1001/jamanetworkopen.2023.28347)
Supplement: Supplement 2. — Data Sharing Statement [file jamanetwopen-e2328347-s002.pdf]

Data Sharing Statement

Chami. Referring and Specialist Physician Gender and Specialist Billing. *JAMA Netw Open*. Published August 25, 2023. doi:10.1001/jamanetworkopen.2023.28347

Data
Data available: No

Additional Information
Explanation for why data not available: We use administrative health data under protection of provincial privacy legislation.
